# GC–MS and molecular docking analyses of phytochemicals from the underutilized plant, *Parkia timoriana* revealed candidate anti-cancerous and anti-inflammatory agents

**DOI:** 10.1038/s41598-022-07320-2

**Published:** 2022-03-01

**Authors:** Laldinfeli Ralte, Laldinliana Khiangte, Nurpen M. Thangjam, Awadhesh Kumar, Y. Tunginba Singh

**Affiliations:** 1 Department of Botany, Mizoram University, Aizawl, Mizoram 796004 India; 2 Department of Horticulture, Aromatic and Medicinal Plants, Mizoram University, Aizawl, Mizoram 796004 India

**Keywords:** Chemical biology, Drug discovery, Plant sciences

## Abstract

Plants are excellent sources of functionally bioactive compounds and essential nutrients. The phytochemical constituents have enormous potential in treating both plant and human diseases. *Parkia timoriana* (Yongchak/Zawngtah), one of the most important underutilized plants popularly consumed in Manipur and Mizoram states of Northeastern region of India, is known for its ethnobotanical and ethnomedicinal values. A significant DPPH (2,2-diphenyl-1-picrylhydrazyl), ABTS (2,2’-azino-bis (3-ethylbenzothiazoline-6-sulfonic acid)), and Phosphomolybdate scavenging activity corresponding to high antioxidant potentials was shown by the extracts from different edible parts of *P. timoriana*. *P. timoriana* extract showed significant antibacterial potential against *Bacillus pumilus*, *Bacillus subtillis*, *Escherichia coli* and *Pseudomonas aeruginosa*. Fourier transform infrared spectroscopy and gas chromatography-mass spectrometry (GC–MS) analyses of the extracts revealed the functional groups and bioactive compounds present in different edible parts of the plant. Characteristic peaks of phenols, carboxylic acids, alkenes, glycogen, alkyl halides, halogen, aliphatic amines, primary and secondary amines, esters, ether, aromatics, lipids, triglycerides, nitro compounds that had antimicrobial, anti-cancer and anti-inflammatory properties etc. were observed. The GC–MS analysis also revealed the occurrence of 49 bioactive compounds that are known to possess a variety of pharmacological activities. Subsequently, in silico molecular docking studies of the identified bioactive compounds predicted potential anticancer and anti-inflammatory properties. To the best of our knowledge, this is the first-hand report on the bioactive compounds of edible parts of *P. timoriana* extracts showing antioxidant, antimicrobial and pharmacological significance. This study can lead to the production of new herbal medicines for various diseases employing *P. timoriana* and perhaps leading to the creation of new medications.

## Introduction

Plants, not only provide essential nutrients for humans, but also provide biologically active compounds that are beneficial for the human health and treatment of various diseases^1^. They contain a wide range of compounds like lipids, phytochemicals, pharmaceutics, flavors and fragrance and have been used in food, pharmaceutical and cosmetics industries etc. In developing countries like India, the use of traditional medicines are the primary source of the health care system and the presence of phytochemical compounds are the main reasons for the use of plants in the traditional practice of medicines^2^. In India, especially in the rural northeastern (NE) region, the use of traditional medicine is a common practice that may be due to the availability of diverse vegetation and the socio-economic conditions of the people of the region^3^. Medicinal plants have a wide range of bioactive compounds that have antimicrobial, anticancer, anti-inflammatory and antioxidant potentials. Plant-derived medicines are frequently prepared from crude extracts, which include a complex combination of various phytochemicals and are utilized to treat both chronic and infectious diseases^4^. Various plant species contain a vast pool of secondary metabolites but only a few of them have been studied and proven to be a substantial source of bioactive compounds. The development of acceptable screening procedures is critical in the search for novel chemicals as well as in quality control^5^. The extraction and characterization of these bioactive compounds have resulted in the delivery of specific medications with a high-activity profile^6^. Fourier-transformed infrared (FTIR) and Gas chromatography-mass spectrometry (GC–MS) have been widely used for observation of functional groups and identification of various bioactive compounds present in plants^7,8^. GC–MS is a reliable technique for the identification of various compounds such as alkaloids, flavonoids, organic acids, amino acids etc. from plant extracts^9^. Also, computer-based tools have evolved as sophisticated drug discovery approaches that may be used to screen medicines from bioactive compounds present in medicinal plants^10^. Computational prediction models are utilized in the in silico prediction of pharmacological, pharmacokinetic and toxicological production and play a crucial role in the selection of procedure leading to pharmaceutical and technological advancement^11^. Molecular docking is an efficient and low-cost approach for creating and testing pharmaceuticals. This technique gives the knowledge on drug-receptor interactions that may be used to anticipate how the drug model will bind to the target proteins^12^ leading to reliable binding at the binding sites of ligands^13^.

*Parkia timoriana* (DC) Merr., commonly known as tree bean belonging to the Fabaceae family, is an underutilized medicinal plant well distributed in Southeast Asia and Northeast (NE) India^14^. *P.timoriana* is a lesser-known nutritious leguminous tree that grows and found abundant in NE region of India, grown in wild, jhum land and backyard of houses and is a multipurpose plant having significant economic importance which includes vegetables, medicine, firewood and ecological significance^15^. *P. timoriana* (Yongchak in Manipuri and Zawngtah in Mizo) is popularly consumed particularly in Manipur and Mizoram states of NE India for its ethnobotanical and ethnomedicinal values.This plant grows in different conditions from colder and hilly regions to warm and foothill regions without special care^16^. It is an ethnobotanically important plant and has high nutritional and medicinal value. The decoctions of bark and fruits are normally used to treat various ailments^17^. The plant is also rich in protein, minerals, essential amino acids, fatty acids, oleic acids, linoleic acids and can be a good source of various other nutrients and supplements^18^. It was also reported that *P. timoriana* had high antioxidant potentials and a good amount of phytochemicals that could have a significant role in anti-cancer, antibacterial and anti-ageing activities^19^. Very few research on the ethnomedicinal^15,20^, preliminary pharmacological^20–22^, biotechnological applications and mass productions^16^ and nutritional composition^23,24^ of *P. timoriana* have been reported so far.

However, to the best of our understanding, there is no report characterizing the presence and evaluation of various bioactive compounds in different edible parts of *P. timoriana*. Therefore, the present study focused on the identification of bioactive compounds from methanolic extracts of flower, capitulum, pod and seed of *P. timoriana* by FTIR and GC–MS analyses. The study also tested the antimicrobial and antioxidant properties of the extracts. Following this, an in silico molecular docking was also performed to identify putative bioactive compounds with anticancer and anti-inflammatory potentials. This study will aid in the prediction of the structures and formulae of bioactive compounds present in *P. timoriana* that could be essential for the discovery and designing of new drugs formulations.

## Results

### Ethnobotanical studies

**Figure 1:**
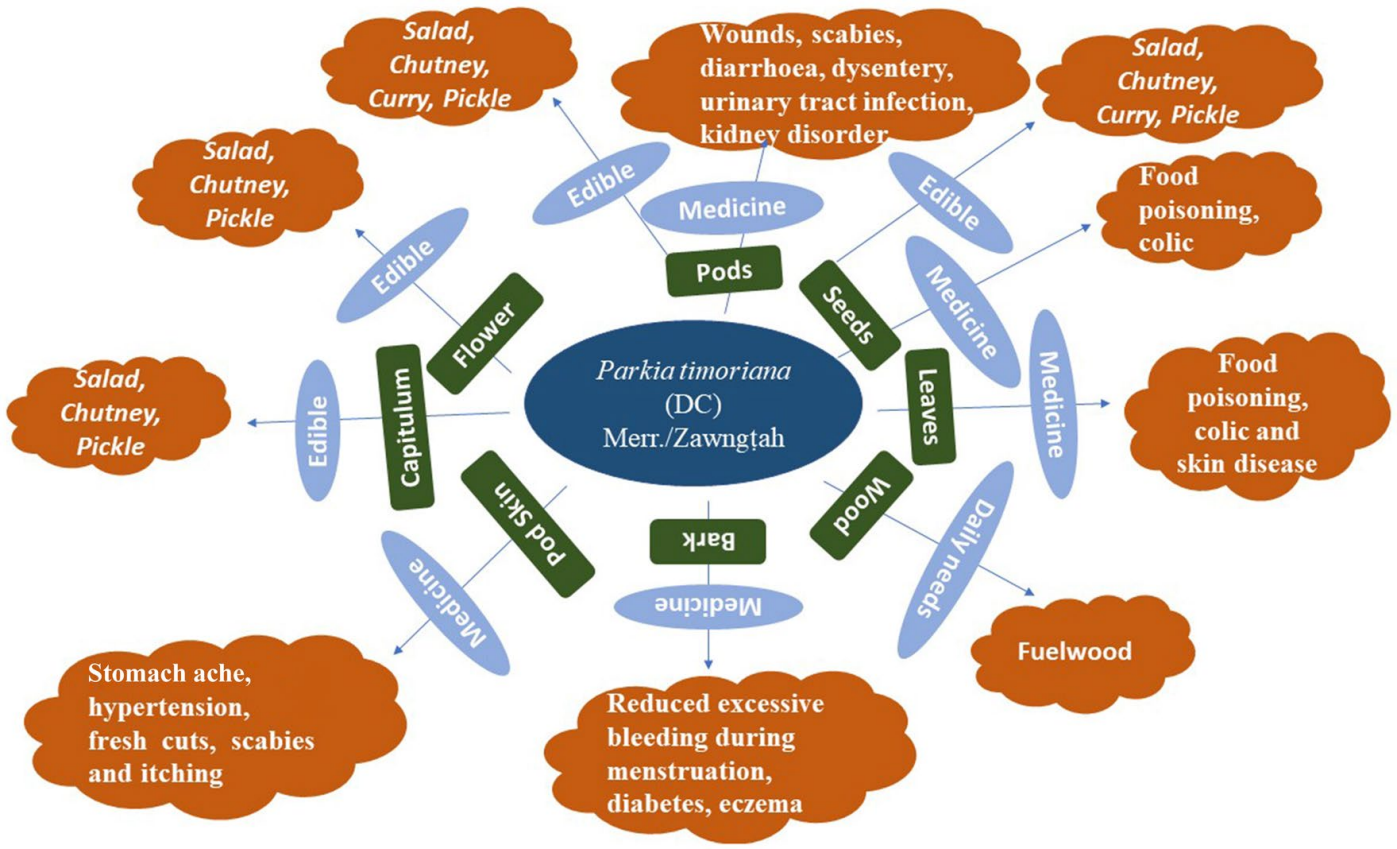
Diagrammatic representation of ethnobotanical uses of *Parkia timoriana.*

Plants are valuable sources of beneficial bioactive compounds for the manufacture of novel chemotherapeutic agents^25^. Investigation on the possibility of using pharmacologically active compounds derived from medicinal plants is an important procedure^26^. It is believed that about 80% of the world's population use medicinal plants due to their high efficacy, low cost, non-narcotic origin and fewer side effects^27^. Various food crops are used for their application in healthcare and therefore are called "medicinal foods". *P. timoriana*, tree bean is an ethnobotanically important plant having high nutritional and medicinal values (Fig. 1).

### Preliminary screening

A preliminary phytochemical screening was done using methanolic extracts of edible parts of *P. timoriana* and the result is shown in Table 1.

**Table 1:** Phytochemical screening of extracted plant parts *P. timoriana.*

Sl No	Name	Alkaloids	Flavonoids	Saponin	Tannins	Terpenoids
1	Capitulum	+	+	+	+	+
2	Flower	+	+	+	+	+
3	Pod	+	+	+	+	+
4	Seed	+	+	+	+	+

The study showed the presence of tannins, terpenoids, phenol, flavonoids, alkaloids in *P. timoriana*. The presence of phytochemicals from this plant had also been reported previously.

### Total flavonoid content

The quantitative phytochemical composition of flower, capitulum, tender pods and seeds of *P. timoriana* are shown in Table 2. Total flavonoid was found to be the highest in the pod (58.38 ± 0.001 mg/g), while flower (28.95 ± 0.002 mg/g) contained the least amount. Previously, it was shown that the methanolic extracts of pods and seeds contained 5.28 mg QE/g and 20.3 mg QE/g respectively^28^ and 15.47 mg CA/g in pods^29^. Tapan^30^ has shown that the methanol extract of pods contained 4.05 mg/g total flavonoid.

**Table 2:** Quantitative phytochemical analysis of *P. timoriana.*

Sl No	Plant parts	Total flavonoid content (mg/g)	Total phenol content (mg/g)
1	Capitulum	36.69 ± 0.002	24.17 ± 0.11
2	Flower	28.95 ± 0.004	18.7 ± 0.161
3	Pod	58.38 ± 0.001	38.21 ± 0.136
4	Seed	35.15 ± 0.002	32.04 ± 0.12

### Total phenol content

The total phenols content of *P. timoriana* is shown in Table 2. The pod had the highest total phenol content of all analyzed samples (38. ± 0.136 mg/g) whereas flower extract had the lowest content (18.7 ± 0.161 mg/g). The result is in agreement with previous reports on *P. timoriana* where pods contained 49.39 mg GAE/g^30^ 36.59 ± 3.80 mg GAE/g^19^.

### Antioxidant assay

#### DPPH Scavenging activity

**Figure 2:**
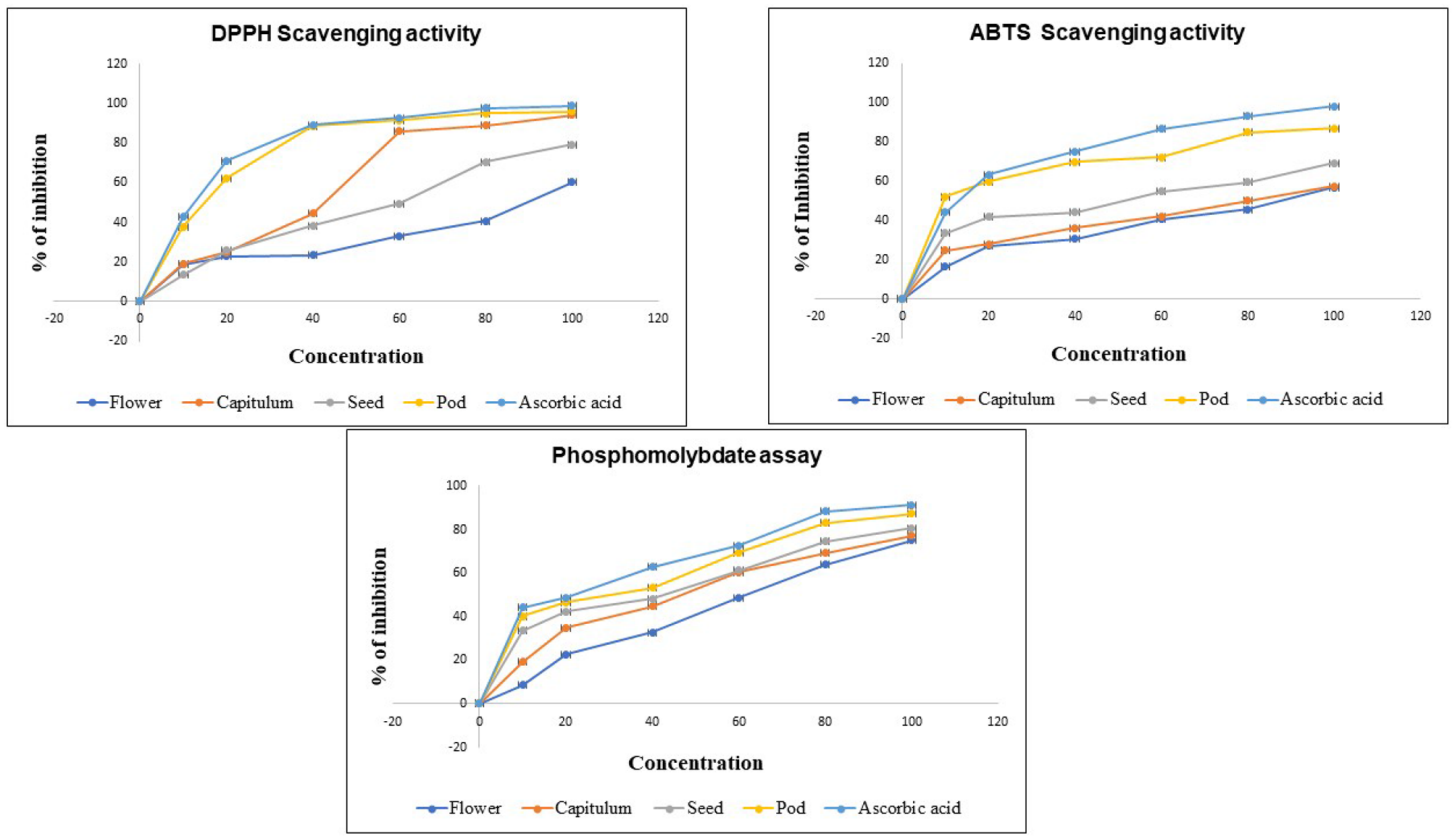
Antioxidant activity of edible parts of *P. timoriana.* (**A**) DPPH scavenging activity; (**B**) ABTS scavenging activity; (**C**) Phosphomolybdate assay.

The antioxidant property of methanolic extract of *P. timoriana* was estimated using DPPH assay. The percentage inhibition of free radical scavenging and IC_50_ is represented in Fig. 2 & and Table 3 respectively. The antioxidant IC_50_ was analogously high in the pod (22.45 µg/ml) and lowest in flower (70.05 µg/ml). Earlier Badu et al.^31^ have reported that the DPPH antioxidant IC_50_ ranged from 160.44 to 157 µg/ml.

**Table 3:** The IC_50_ of different antioxidant assays.

Sl No	Plant parts	DPPH IC_50_ (µg/ml)	ABTS IC_50_ (µg/ml)	Phosphomolybdate IC_50_ (µg/ml)
1	Capitulum	44.25 ± 0.11	77.55 ± 0.3	53 ± 0.14
2	Flower	70.05 ± 0.7	82.78 ± 0.2	63.09 ± 0.1
3	Pod	22.45 ± 0.4	27.55 ± 0.21	38.52 ± 0.09
4	Seed	47.43 ± 0.3	56.63 ± 0.6	46.28 ± 0.1
5	Ascorbic acid	20.51 ± 0.1	24.71 ± 0.3	33.43 ± 0.8

#### ABTS Scavenging activity

All the *P. timoriana* extracts scavenged ABTS radical in a concentration-dependent manner (10–100 µg/ml) (Fig. 2). The ABTS radical scavenging capacity of samples could be ranked as Pod > Seed > Capitulum > Flower. The pod and seed had high ABTS radical scavenging capabilities. The IC_50_ of ABTS scavenging activities ranged from 27.55 µg/ml to 82.78 µg/ml where pod had the highest value of 27.55 µg/ml, which was comparable to that of Ascorbic acid (24.71 µg/ml).

### Phosphomolybdate assay

The quantitative total antioxidant capacity was also assessed using Phosphomolybdate assay. Based on the scavenging activity, the extracts could again be ranked as Pod > Seed > Capitulum > Flower (Fig. 2). The IC_50_ value ranged from 38.52 µg/ml in pod to 63.09 µg/ml in flower. The IC_50_ of the pod is also comparable to that of the standard, Ascorbic acid (33.43 µg/ml). The significant antioxidant potential of the pod, which is statistically comparable to that of standard Ascorbic acid, showed that the extract contained strong antioxidants, which could be due to the presence of phenolic compounds.

### Antibacterial activity

**Figure 3:**
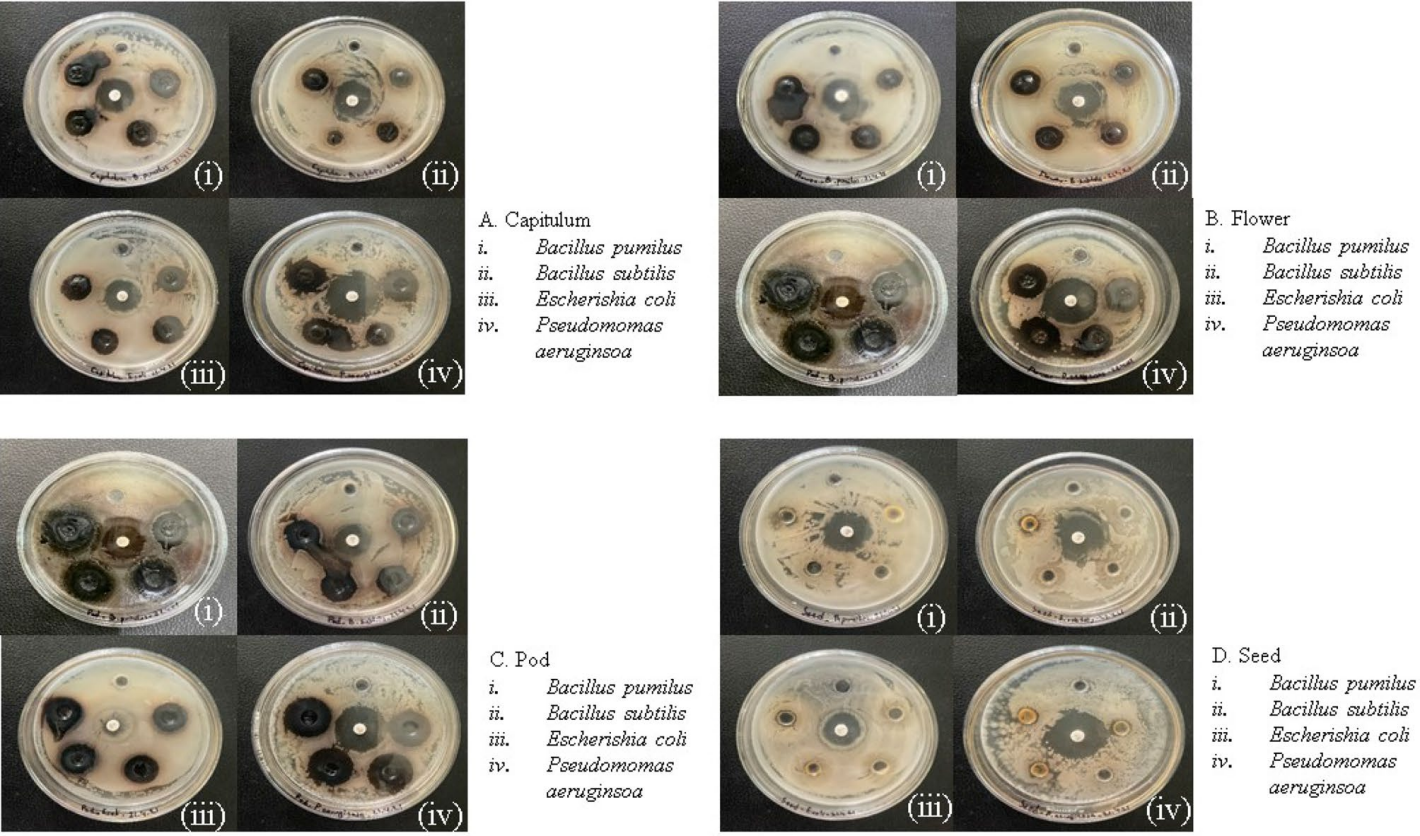
Antibacterial activity of *P. timoriana.*

The present study investigates the antibacterial activity of edible parts of tree bean from Mizoram, India. The result shows that the methanol extracts of *P. timoriana* exhibit antibacterial activities against the four tested bacterial strain (Table 4 and Fig. 3). The diameter of the zones of inhibition ranged from 9 to 17.66 mm, the highest inhibition zone was observed in *E. coli* (17.66 mm), followed by *B. pumilus*, *P. aeruginosa* and *B. subtilis* (15.33 mm, 14.33 mm and 13 mm respectively).

**Table 4:** Antibacterial activity of methanolic extract of *P. timoriana* against the selected bacterial strain.

Sl No	Plant parts used	Inhibition zone (mm)				
		*Bacillus pumilus*	*Bacillus subtilis*	*Escherichia coli*	*Pseudomonas aeruginosa*	*Range*
1	Capitulum	11.33 ± 0.08	10.33 ± 0.35	15.33 ± 0.59	10.66 ± 0.44	High
2	Flower	10.66 ± 0.24	10 ± 0.54	13.66 ± 1.09	10.66 ± 0.05	High
3	Pod	15.33 ± 0.14	13 ± 0.84	17.66 ± 0.18	14.33 ± 0.77	Very High
4	Seed	10.33 ± 0.45	10 ± 0.29	17 ± 0.55	9.33 ± 0.37	High
Control						
Rifampicin		17 ± 0.54	14 ± 0.13	20 ± 0.72	16 ± 0.19	Very High

The quantitative evaluation based on the inhibition growth showed that the pod had very high (*x* > 12 mm) inhibitions against all the tested bacterial strains. Other parts- capitulum, flower and seed also had high (*x* = 8–12 mm) inhibitions zone against all bacterial strains.

### Minimum inhibitory concentration (MIC)

**Figure 4:**
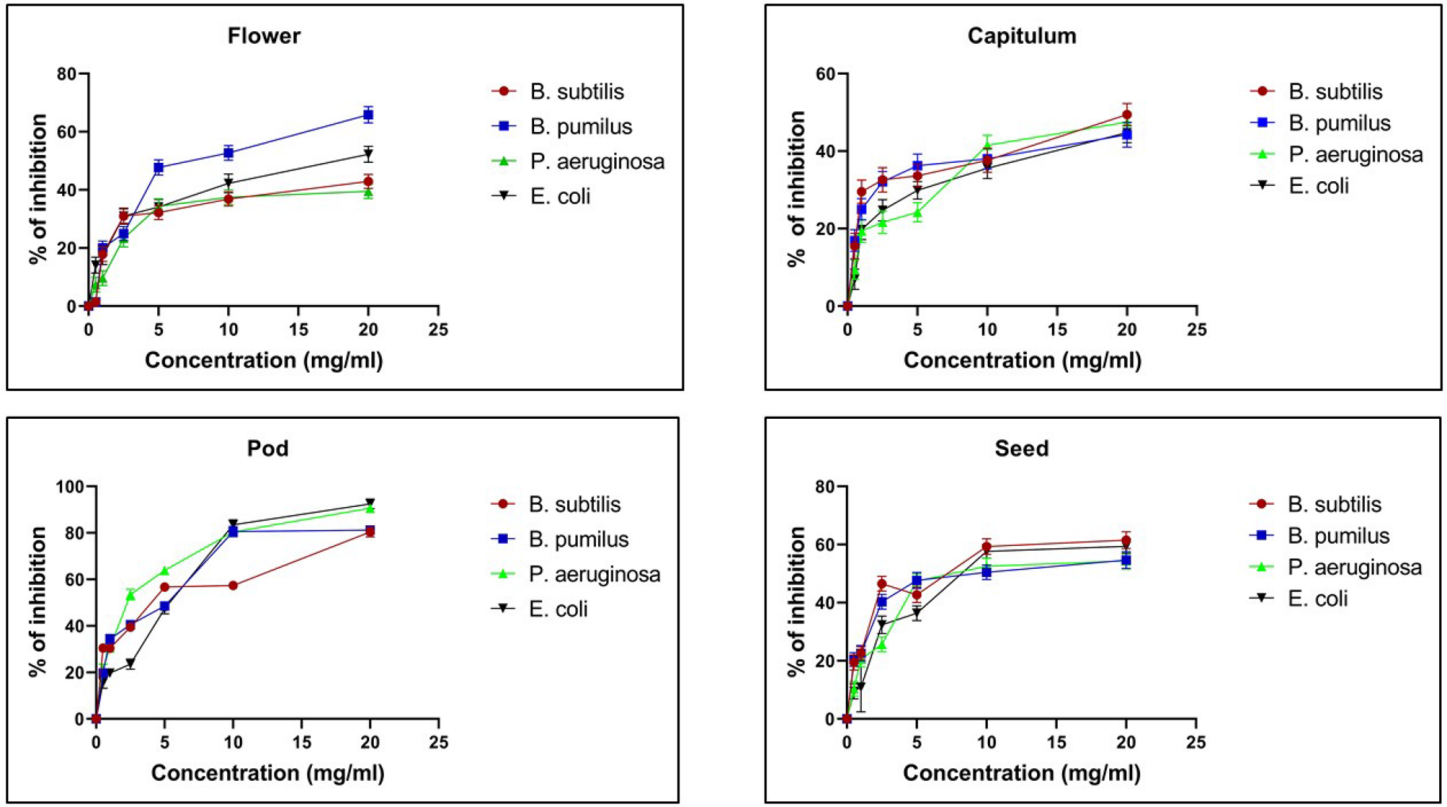
Percentage inhibition showing antibacterial activity of *P. timoriana* extracts against microorganisms, *B. subtilis, B. pumilus, P. aeruginosa* and *E. coli*.

MIC values determined from the extracts varied from 2.19 to 7.3 mg/ml (Table 5). The percentage inhibition of antibacterial activity is shown in Fig. 4. Pod gave MIC values of 3.5 mg/ml, 3.4 mg/ml, and 2.19 mg/ml, and 3.04 mg/ml respectively against *B. subtilis, B. pumilus, E. coli*, and *P. aeruginosa*.

**Table 5:** Minimum inhibition concentration (mg/ml) of methanolic extracts from *P. timoriana*.

Sl. No	Plant part	MIC (mg/ml)			
		*Bacillus subtilis*	*Bacillus pumilus*	*Pseudomonas aeruginosa*	*Escherichia coli*
1	Flower	6.47 ± 0.33	3.24 ± 1.45	6.73 ± 0.27	6.13 ± 0.81
2	Capitulum	6.5 ± 0.59	7.3 ± 0.11	6.69 ± 1.01	6.9 ± 0.43
3	Pod	3.5 ± 0.18	3.4 ± 0.67	3.04 ± 0.32	2.19 ± 0.35
4	Seed	4.47 ± 0.28	5.02 ± 0.47	5.1 ± 0.09	4.15 ± 0.43

### FTIR analysis

**Figure 5:**
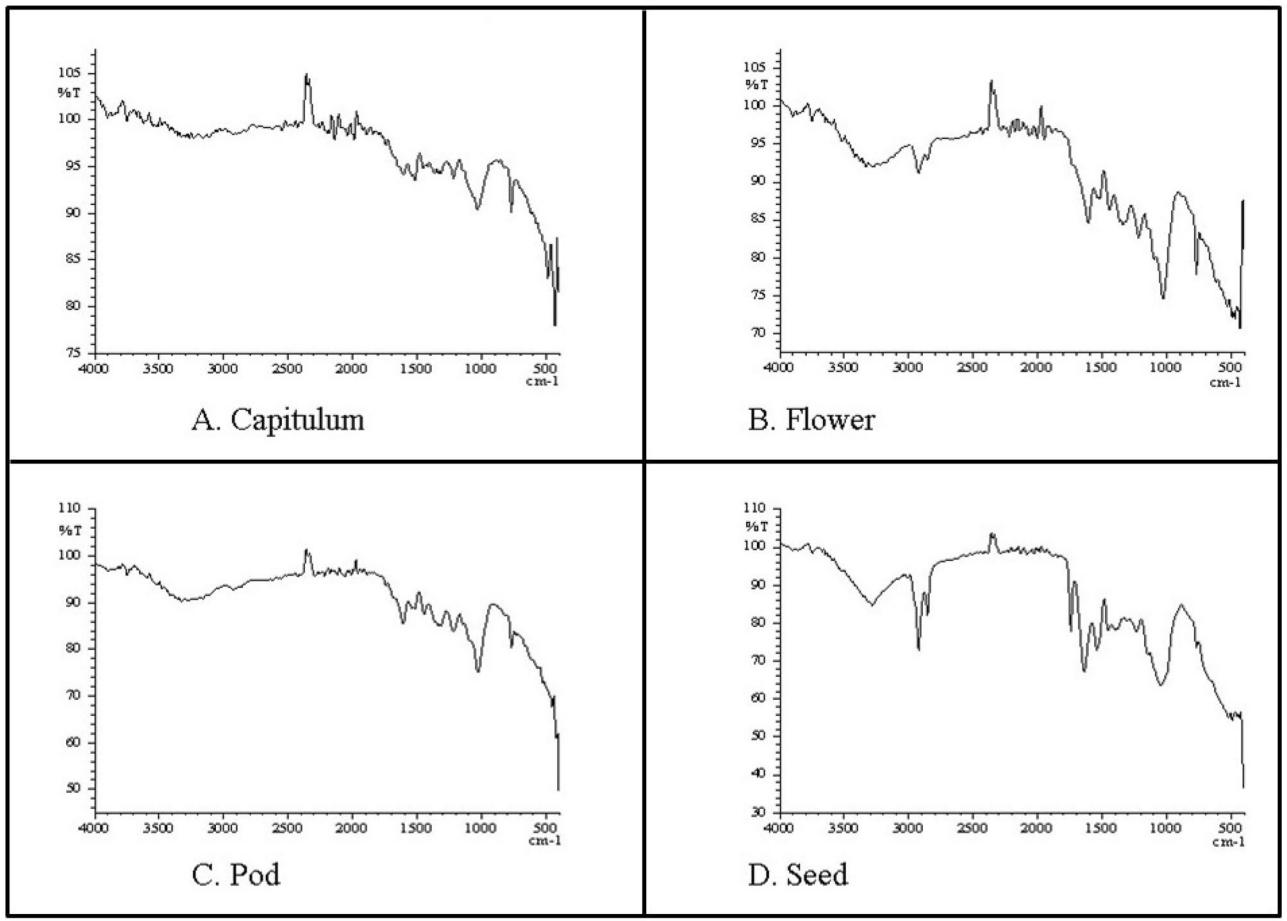
FTIR spectra of edible parts of *P. timoriana.*

FTIR spectroscopy was employed to identify the functional groups of bioactive components present in *P. timoriana* (Table 6 and Fig. 5). FTIR spectrum confirmed the presence of hydroxyl group (O–H), aldehyde (C–H), alkenes (C=C), carboxyl (C=O), nitrogen-containing group (N–O), alkanes (C–C), aromatic primary amine (C–N), amines (N–H), alkynes (C≡C), aliphatic Bromo compounds (C–Br), phenols, carboxylic acids, glycogen, alkyl halides, halogen, aliphatic amines, primary and secondary amines, esters, ether, aromatics, lipids, triglycerides, nitro compounds and these functional groups are the integral parts of various secondary metabolites such as alkaloids, flavonoids, terpenoids, polyphenol and tannins^32^.

**Table 6:** Details of FTIR spectrum of *P. timoriana.*

Frequency range (cm^−1^)	Samples				Functional group
	Flower	Capitulum	Pod	Seed	
3870–3550					O–H stretch alcohol
3500–3200	3332.99			3286.7	O–H stretch vibration presence of alcohols, phenols
3300–2850	2924.09			2924.09	O–H stretch vibration, carboxylic acids
2500–2300					C–H stretch vibration, alkenes
2260–2100					C=C stretch vibration, alkynes
1990–1739				1743.65	Ester C=O stretch, lipid, triglycerides
1700–1600	1604.77	1604.77	1604.77	1643.35	C=C stretch vibration, alkenes
1550–1475	1519.91	1519.91			N–O asymmetric stretch, nitro compounds
1470–1400	1442.75		1450.47	1458.18	C–C stretch vibration, aromatics
1400–1320	1342.46			1396.46	N–O stretch vibration, nitro compounds
1300–1290					C–O stretch vibration, alcohol, carboxylic acids, esters, ether
1275–1150	1219.01	1219.01	1219.01	1234.44	C–H wag stretch vibration, alkyl halides
1020–1000					C-N stretch vibration, aliphatic amines
990–800	871.82	825.53			N–H wag stretch vibration, primary & secondary amines
790–690	771.53	771.53	771.53	771.53	C(triple bond)C-HC-H bend stretch vibration, alkynes
680–510	532.25	617.22	663.51	516.92	C–Br stretch vibration, alkyl halides, glycogen
490–400		486.06	455.2	455.2	Halogen compound

### GC–MS analysis

**Figure 6:**
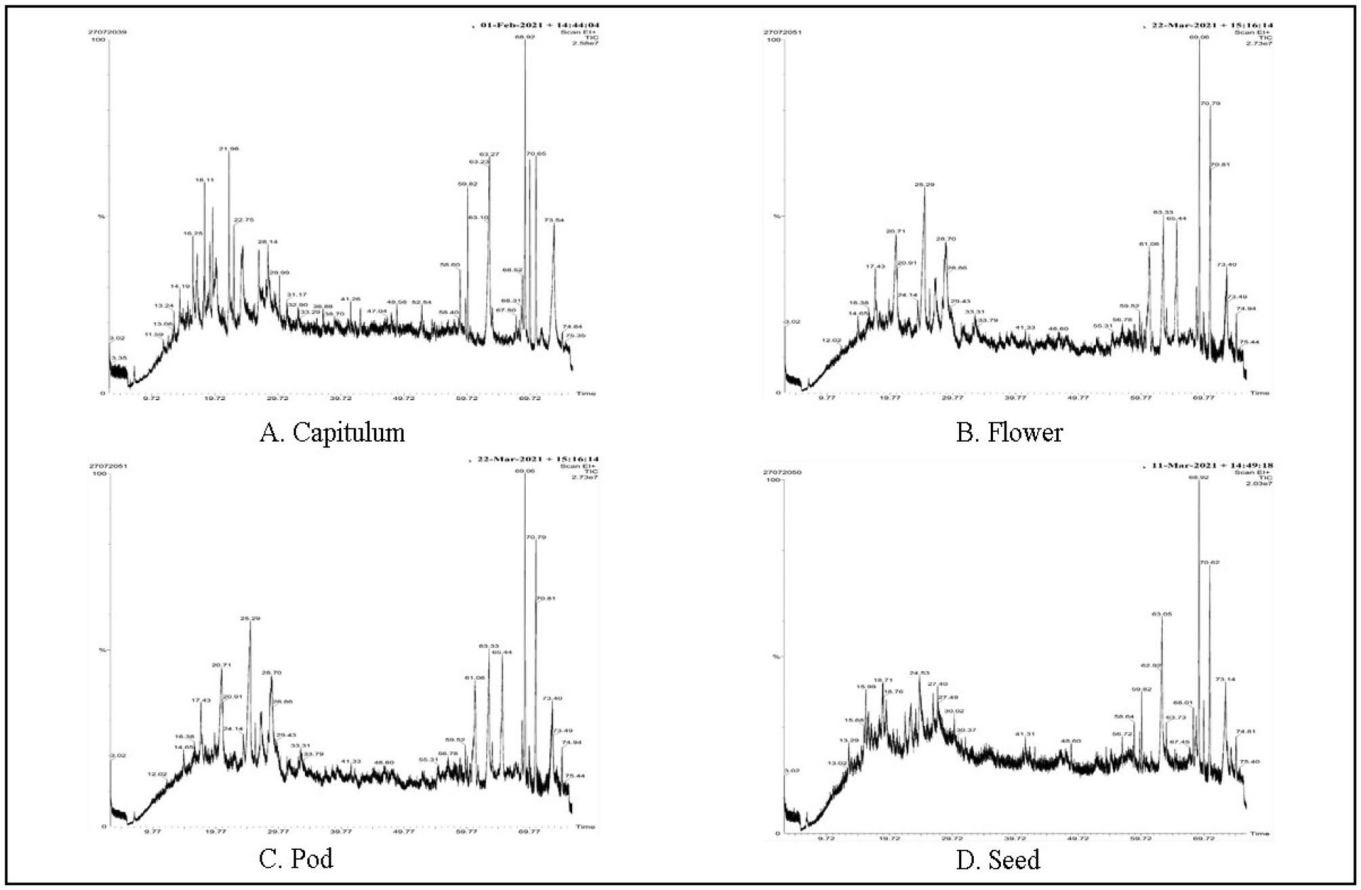
GC–MS chromatogram of edible parts *of P. timoriana* (**A** Capitulum; **B** Flower; **C** Pod; **D** Seed).

Evaluation of the chemical structure and composition of the extracts can designate various biological potential of different medicinal plant extracts. However, to the best of our knowledge, there is no report on GC–MS based plant metabolic characterization to reveal the presence of various bioactive compounds in methanolic extracts of edible parts of *P. timoriana*. Therefore, the GC–MS analysis was performed in a predetermined study. A total of 104 peaks were observed in all plant parts, each peak designated the bioactive compounds that were recorded by relating their peak retention time, molecular weight, molecular formula to that of the known compounds proposed by the NIST library (Table 7). The highest number of compounds (32) was observed in the flower extract followed by pod (26), capitulum (26) and seed (20) (Fig. 6).

**Table 7:** GC–MS analysis of edible parts of *P. timoriana.*

Peak	RT	Molecular Weight	Molecular Formula	Name of the compound	Medicinal uses
**Flower**					
1	12.09	140.22	C9H16O	Cyclopentanone, 2-(1- Methylpropyl) -	Anti-inflammatory, analgesic, anticonvulsant^33^
2	13.29	164.16	C6H12O5	Beta –L-Arabinopyranoside, Methyl	Catechol-O-methyltransferase inhibitor, methyl donor Methylguanidine inhibitor, 17-beta-hydroxysteroid dehydrogenase inhibitor, beta-adrenergic receptor blocker Beta-adrenergic agents, anticancer (liver, lung, breast, and prostate), antioxidant (LDL)^34^
3	14.94	138.16	C8H10O2	3-Methyl-2-(2-Oxopropyl) Furan	Antioxidant, antimicrobial and bacteriocide, Antipyretic, antiinflammatory activity^35^
4	15.95	130.18	C7H14O2	Heptanoic acid	No activity reported
5	17.87	138.16	C8H10O2	3-Methyl-2-(2-Oxopropyl) Furan	Antioxidant, antimicrobial and bacteriocide activity. Antipyretic, antiinflammatory activity^35^
6	18.73	144.21	C8H16O2	Octanoic acid	Antibacterial activity
7	19.83	204.33	C10H20O2S	3-N-Hexylthiolane, S,S-Dioxide	Anti-tumour, anti-cancer, anti-dote, antimicrobial and anti-viral activity^35^
8	20.43	172.26	C10H20O2	N-Decanoic acid	Antibacterial and antifungal activity^35^
9	21.53	322.5	C21H38O2	11,14-Eicosadienoic acid, Methyl ester	Anti-inflammatory, anti-oxidant, anti-arthritic, anti-coronary^35^
10	22.3	158.24	C9H18O2	Nonanoic acid	Anti-microbial, anti-inflammatory, antitumor, anti hyperpigmentative, antiproliferative, anti-acne, cyto toxic, Anti-leukemic, oxy radical scavenging Activity^35^
11	24.53	170.24	C10H18O2	2(3H)-Furanone, 5Hexyldihydro -	Aromatic compounds
12	27.02	172.26	C10H20O2	N-Decanoic acid	Antibacterial and antifungal activity^35^
13	31.16	268.47	C18H36O	Oxirane, hexadecyl	Antimicrobial, adhesive^35^
14	42.81	530.9	C37H70O	Z,Z-6 , 28-Heptatriactontadien-2-one	Vasodilator^36^
15	46.79	290.9	C16H31ClO2	Chloroacetic acid, Tetradecyl ester	Antioxidant, antimicrobial and bacteriocide, anti- inflammatory activity^35^
16	47.71	168.19	C9H12O3	4-Methoxy-6-Methyl-6,7-dihydro-4H-Furo [3,2-c ] Pyran	No activity reported
17	48.61	422.8	C29H58O	Nonacosanal	Antihypersensitive, vasodilator, angiotensin AT2 receptor antagonist and saluretic^36^
18	52.78	282.5	C18H34O2	Oleic acid	Antimicrobial, Antifungal, anticonvulsive activity, Antiadhesive, Antiallergic, Antianalgesic, Antiatherosclerosis, Anesthetic, Antihelmenthic, Antianxiety, Antibacterial, Antiberiberi, Antibiotic, Anticancer, Anticonvulsant, Antidiabetic, Antidiarrheic, Antifertility, Antigastric, Anti-inflammatory, Antiobesity, Antioxidant, Antiulcer, Antitubercellosic, Anticold, Antihepatotoxic and Antiviral activityanemiagenic, dermatitigenic^37^
19	57.84	366.7	C25H50O	2-Pentacosanone	No activity reported
20	58.63	366.7	C25H50O	2-Pentacosanone	No activity reported
21	59.49	324.58	C22H44O	13-Docosen-1-ol, (Z) -	No activity reported
22	59.82	298.5	C19H38O2	Methyl Stearate	GABA aminotransferase inhibitor, anti-inflammatory, intestinal lipid metabolism regulator, antihelmintic, antinociceptive^38^
23	60.99	254.41	C16H30O2	Palmitoleic acid	Anti-inflammatory, essential oils
24	63.7	312.5	C20H40O2	Eicosanoic acid	Reduced heart diseases, kidney and liver function, blood clotting
25	65.39	286.92	C18H35Cl	Cis-1-chloro-9-octadecene	No activity reported
26	68.58	198.3	C12H22O2	7-Decyloxepan-2-one	No activity reported
27	69.07	254.41	C16H30O2	Palmitoleic acid	Anti-inflammatory, essential oils
28	69.69	298.5	C19H38O2	Methyl Stearate	GABA aminotransferase inhibitor, anti-inflammatory, intestinal lipid metabolism regulator, antihelmintic, antinociceptive^38^
29	70.82	546.8	C32H67O4P	Dihexadecyl Phosphate	Emulsifying agent
30	74.02	340.6	C22H44O2	Docasanoic acid	Antibacterial^38^
31	74.92	282.5	C18H34O2	6-Octadecenoic acid	Antimicrobial activity, anti-cancer^39^
**Capitulum**					
1	13.24	140.22	C9H16O	Cyclopentanone, 2-(1- Methylpropyl) -	Anti-inflammatory, analgesic, anticonvulsant^33^
2	14.19	164.16	C6H12O5	Beta-L-Arabinopyranoside, Methyl	Catechol-O-methyltransferase inhibitor, methyl donor Methylguanidine inhibitor, 17-beta-hydroxysteroid dehydrogenase inhibitor, beta-adrenergic receptor blocker Beta-adrenergic agents, anticancer (liver, lung, breast, and prostate), antioxidant (LDL)^34^
3	16.25	138.16	C8H10O2	3-Methyl-2-(2-Oxopropyl) Furan	Antioxidant, antimicrobial and bacteriocide, Antipyretic, antiinflammatory activity^35^
4	16.94	130.18	C7H14O2	Heptanoic acid	Used in the preparation of esters for the fragrance industry, and as an additive in cigarettes
5	18	126.2	C8H14O	5, 5-Dimethyl-Cyclohex-3 en-1-ol	No activity reported
6	18.11	134.22	C10H14	P-Mentha-1,5,8-Triene	No activity reported
7	18.97	152.23	C10H16O	Hotrienol	Prevent/reduce metabolic abnormalities and various diseases^40^
8	19.39	188.34	C9H20O2Si	Cyclohexylmethyl silane	No activity reported
9	19.92	144.21	C8H16O2	Octanoic acid	Antibacterial activity
10	21.98	234.33	C15H22O2	Myrtenyl angelate	Antimicrobial, anti-inflammatory, antioxidants and anti-termitic activities^40^
11	22.75	152.23	C10H16O	1-Cyclohexane-1-Methanol, 4-(1-Methyl ethenyl)—, Formate	Antioxidant activity
12	24.13	172.26	C10H20O2	N-Decanoic acid	Antibacterial and antifungal activity^35^
13	26.71	254.41	C16H30O2	Valeric acid, Undec-2- enyl ester	Used in the synthesis of its esters. Volatile esters of Valeric acid have pleasant odours which are used in perfumes and cosmetics
14	28.14	530.9	C37H70O	Z,Z-6 , 28-Heptatriactontadien-2-one	Vasodilator^36^
15	29.99	204.33	C10H20O2S	3-N-Hexylthiolane, S,S-dioxide	Anti-tumour, anti-cancer, anti-dote, antimicrobial and anti-viral activity^36^
16	48.58	366.7	C25H50O	Pentacosanal	No activity reported
17	52.54	462.6	C25H42N4O4	2-Nonadecanone 2,4—Dinitrophenylhydrazine	Antimicrobial^40^
18	58.6	366.7	C25H50O	2-Pentacosanone	No activity reported
19	59.47	422.8	C29H58O	Nonacosanal	Antihypersensitive, vasodilator, angiotensin AT2 receptor antagonist and saluretic^36^
20	59.82	228.37	C14H28O2	Methyl 11-Methyl-Dodecanoate	Anti-fungal, antioxidant activities^35^
21	63.27	312.5	C20H40O2	Eicosanoic acid	Reduced heart diseases, kidney and liver function, blood clotting
22	68.52	254.41	C16H30O2	Palmitoleic acid	Anti-inflammatory, essential oils
23	68.92	282.5	C18H34O2	Oleic acid	Antimicrobial, Antifungal, anticonvulsive activity, Antiadhesive, Antiallergic, Antianalgesic, Antiatherosclerosis, Anesthetic, Antihelmenthic, Antianxiety, Antibacterial, Antiberiberi, Antibiotic, Anticancer, Anticonvulsant, Antidiabetic, Antidiarrheic, Antifertility, Antigastric, Anti-inflammatory, Antiobesity, Antioxidant, Antiulcer, Antitubercellosic, Anticold, Antihepatotoxic and Antiviral activityanemiagenic, dermatitigenic^37^
24	69.66	298.5	C19H38O2	Methyl Stearate	GABA aminotransferase inhibitor, anti-inflammatory, intestinal lipid metabolism regulator, antihelmintic, antinociceptive^38^
25	70.65	546.8	C32H67O4P	Dihexadecyl Phosphate	Emulsifying agent
26	73.54	340.6	C22H44O2	Docasanoic acid	Antibacterial^41^
**Pod**					
1	14.65	164.16	C6H12O5	Beta –LArabinopyranoside, Methyl	Catechol-O-methyltransferase inhibitor, methyl donor Methylguanidine inhibitor, 17-beta-hydroxysteroid dehydrogenase inhibitor, beta-adrenergic receptor blocker Beta-adrenergic agents, anticancer (liver, lung, breast, and prostate), antioxidant (LDL)^34^
2	16.38	214.38	C14H30O	Heptane, 1,1- oxybis	Antibacterial, antioxidant activities
3	17.43	130.18		Heptanoic acid	Used in the preparation of esters for the fragrance industry, and as an additive in cigarettes
4	19.58	170.25	C7H14O2	9—Decenoic acid	Antimicrobial activity^39^
5	20.71	144.21	C10H18O2	Octanoic acid	Antibacterial activity
6	24.14	184.31	C8H16O2	Oxirane, Decyl	Antimicrobial, adhesive^39^
7	25.29	158.24	C12H24O	Nonanoic acid	Anti-microbial, anti-inflammatory, antitumor, anti hyperpigmentative, antiproliferative, anti-acne, cyto toxic, Anti-leukemic, oxy radical scavenging Activity^40^
8	26.07	342.65	C9H18O2	1,2,4-Benzenetriol, Tris ( Trimethyl silyl) Ether	Antibacterial, anti-infective
9	26.89	126.2	C15H30O3Si3	5, 5-Dimethyl-Cyclohex-3 en-1-ol	No activity reported
10	28.7	172.26	C8H14O	N-Decanoic acid	Antibacterial and antifungal activity^35^
11	33.31	530.9	C10H20O2	Z,Z-6 , 28-Heptatriactontadien-2-one	Vasodilator^36^
12	55.31	128.21	C37H70O	4-Methyl-6-Hepten-4-olide	No activity reported
13	56.78	166.13	C8H16O	Phthalic acid	Antioxidant activity and larvicidal activities^36^
14	58.68	366.7	C8H6O4	2-Pentacosanone	No activity reported
15	59.52		C25H50O	Oleyl alcohol, Cholodifluoroacetate	Used as a nonionic surfactant, emulsifier, emollient and thickener in skin creams, lotions and other cosmetic products including shampoos
16	59.88	298.5		Methyl Stearate	GABA aminotransferase inhibitor, anti-inflammatory, intestinal lipid metabolism regulator, antihelmintic, antinociceptive^38^
17	61.06	282.5	C19H38O2	6-Octadecenoic acid	Antimicrobial activity, anti-cancer^39^
18	63.33	652.9	C18H34O2	L-( +)-Ascorbic acid, 2,6Dihexadecanoate	Antioxidant and reduces the triglycerides level—Protects LDL against peroxidation and inhibits the progression of atherosclerosis, Antiallergic, Antianemic, Antianxiety, Antibacterial, Antibronchitic, Anticancer, Anticarcinogenic, Anticataract, Anticoagulant, Anticonvulsant, Antidiabetic, Antidiarrheic, Antifatigue, Antifertility, Antigastric, Anti-inflammatory, Antimalarial, Antioxidant, Antistress, Antiulcer, Antiatheroscelerotic, Anticold, Antiglaucomic, Antihepatic, Antihypertensive, Antiplague, Antiproliferant, Antiprotozoal, Antiseptic, Antistroke, Antituberculic, Antitumer, CNSStimulant, Chelator, Chemopreventive, CytochromeP450Inducer, Deodorant, Dermal, Detoxicant, Flavor, Hypolipidimic, Neuroprotective, Neurotransmitter, Termiticide and Antiviral activity^37^
19	63.82	254.41	C38H68O8	Palmitoleic acid	Anti-inflammatory, essential oils
20	65.44	546.8	C16H30O2	Dihexadecyl Phosphate	Emulsifying agent
21	68.58	198.3	C32H67O4P	7-Decyloxepan-2-one	No activity reported
22	69.06	254.41	C12H22O2	Palmitoleic acid	Anti-inflammatory, essential oils
23	69.72	410.71	C16H30O2	Heptacosanoic acid, 25-Methy—, Methyl ester	Antimicrobial^41^
24	70.79	546.8	C27H54O2	Dihexadecyl Phosphate	Emulsifying agent
25	73.4	340.6	C32H67O4P	Docasanoic acid	Antibacterial^41^
26	74.94	254.41	C22H44O2	Palmitoleic acid	Anti-inflammatory, essential oils
**Seed**					
1	13.29	164.16	C6H12O5	Beta –L-Arabinopyranoside, Methyl	Catechol-O-methyltransferase inhibitor, methyl donor Methylguanidine inhibitor, 17-beta-hydroxysteroid dehydrogenase inhibitor, beta-adrenergic receptor blocker Beta-adrenergic agents, anticancer (liver, lung, breast, and prostate), antioxidant (LDL)^34^
2	15.68	188.34	C9H20O2Si	Cyclohexylmethyl silane	No activity reported
3	15.98	130.18	C7H14O2	Heptanoic acid	Used in the preparation of esters for the fragrance industry, and as an additive in cigarettes
4	18.71	144.21	C8H16O2	Octanoic acid	Antibacterial activity
5	22.23	170.25	C10H18O2	Cyclohexane butanoic acid	Effect on CNS, antioxidant, analgesic and anti-inflammatory activity^42^
6	23.1	282.5	C18H34O2	Oleic acid	Antimicrobial, Antifungal, anticonvulsive activity, Antiadhesive, Antiallergic, Antianalgesic, Antiatherosclerosis, Anesthetic, Antihelmenthic, Antianxiety, Antibacterial, Antiberiberi, Antibiotic, Anticancer, Anticonvulsant, Antidiabetic, Antidiarrheic, Antifertility, Antigastric, Anti-inflammatory, Antiobesity, Antioxidant, Antiulcer, Antitubercellosic, Anticold, Antihepatotoxic and Antiviral activityanemiagenic, dermatitigenic^37^
7	24.53	170.21	C8H14N2O2	1H-Imidazole, 2-(Diethoxy methyl) -	Antimycobacterial activity, anticancer, antifungal, analgesic and anti-HIV activities^35^
8	26.67	172.26	C10H20O2	N-Decanoic acid	Antibacterial and antifungal activity^35^
9	27.4	530.9	C37H70O	Z,Z-6 , 28Heptatriactontadien-2-one	Vasodilator^36^
10	58.61	366.7	C25H50O	2-Pentacosanone	No activity reported
11	59.49	422.8	C29H58O	Nonacosanal	Antihypersensitive, vasodilator, angiotensin AT2 receptor antagonist and saluretic^36^
12	59.82	298.5	C19H38O2	Methyl stearate	GABA aminotransferase inhibitor, anti-inflammatory, intestinal lipid metabolism regulator, antihelmintic, antinociceptive^38^
13	63.05	322.6	C23H46	5-methyl-Z-5-Docosene	No activity reported
14	68.01	280.44	C18H32O2	9,12-Octadecadienoic acid (Z,Z) -	Antiinflammatory, hypocholesterolemic, cancer preventive,
					hepatoprotective, nematicide, insectifuge antihistaminic,
					antiarthritic, anticoronary, antieczemic antiacne, 5-Alpha
					reductase inhibitor Antiandrogenic^38^
15	68.47	198.3	C12H22O2	2-Oxepanone, 7-Hexyl -	Used as a food flavouring agent
16	68.92	254.41	C16H30O2	Palmitoleic acid	Anti-inflammatory, essential oils
17	69.66	298.5	C19H38O2	Methyl stearate	GABA aminotransferase inhibitor, anti-inflammatory, intestinal lipid metabolism regulator, antihelmintic, antinociceptive^38^
18	70.62	546.8	C32H67O4P	Dihexadecyl Phosphate	Emulsifying agent
19	73.14	322.6	C23H46	5-methyl-Z-5-Docosene	Antibacterial, antidiabetic, antitumour activities^39^
20	74.81	282.5	C18H34O2	6-Octadecenoic acid	Antimicrobial activity, anti-cancer^39^

### Molecular docking

**Figure 7:**
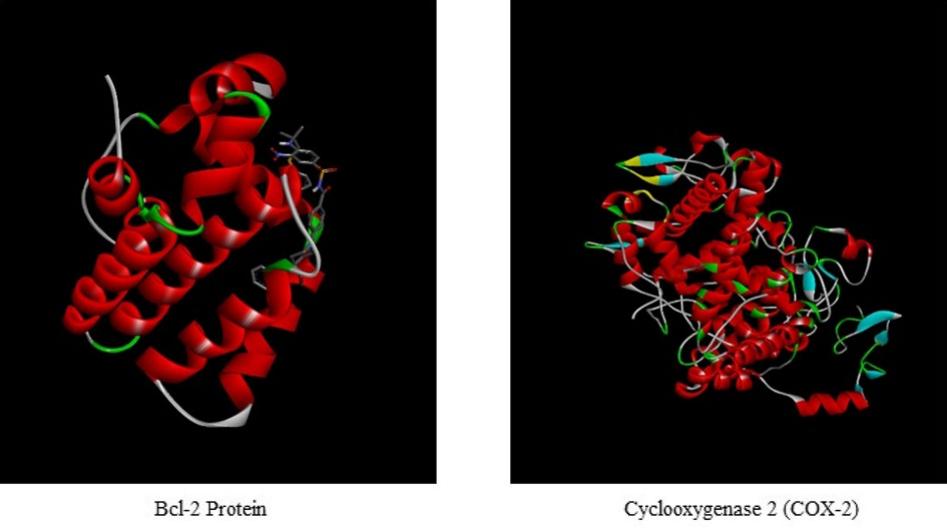
The target Protein Bcl-2 and Cyclooxygenase 2 (COX-2). (Photo Courtesy: Protein DataBank, www.rcsb.org).

Further, six bioactive compounds identified by GC–MS analysis from *P. timoriana* were selected and subjected to molecular docking with BCL-2 and COX-2 proteins (Fig. 7). Paclitaxol (for anticancer) and Oxitasim (anti-inflammatory) were used as standard controls. The 2D structures of bioactive compounds were first retrieved from the PubChem database. These compounds were selected based on Lipinski’s rule of five parameters such as molecular weight, log *P*, number of hydrogen bond donors and number of hydrogen bond acceptors (Table 8).

**Table 8:** Lipinski’s properties of the selected bioactive compounds from extracts of *P. timoriana.*

Sl. No	Compound name	Molecular weight (< 500kD)	Log P (< 5)	H-bond donor (< 5)	H-bond acceptor (< 10)
1	Methyl beta-L-arabinopyranoside	164.16 g/mol	− 2	3	5
2	3-n-Hexylthiolane, S,S-dioxide	204.33 g/mol	3.1	0	2
3	L-(+)-Ascorbic acid, 2,6-Dihexadecanoate	176.12 g/mol	− 1.6	4	6
4	1H-Imidazole, 2-(Diethoxy methyl) -	214.22 g/mol	0.5	1	5
5	Oleic acid	282.5 g/mol	4.5	1	2
6	Cyclohexane butanoic acid	170.25 g/mol	3.4	1	2

**Figure 8:**
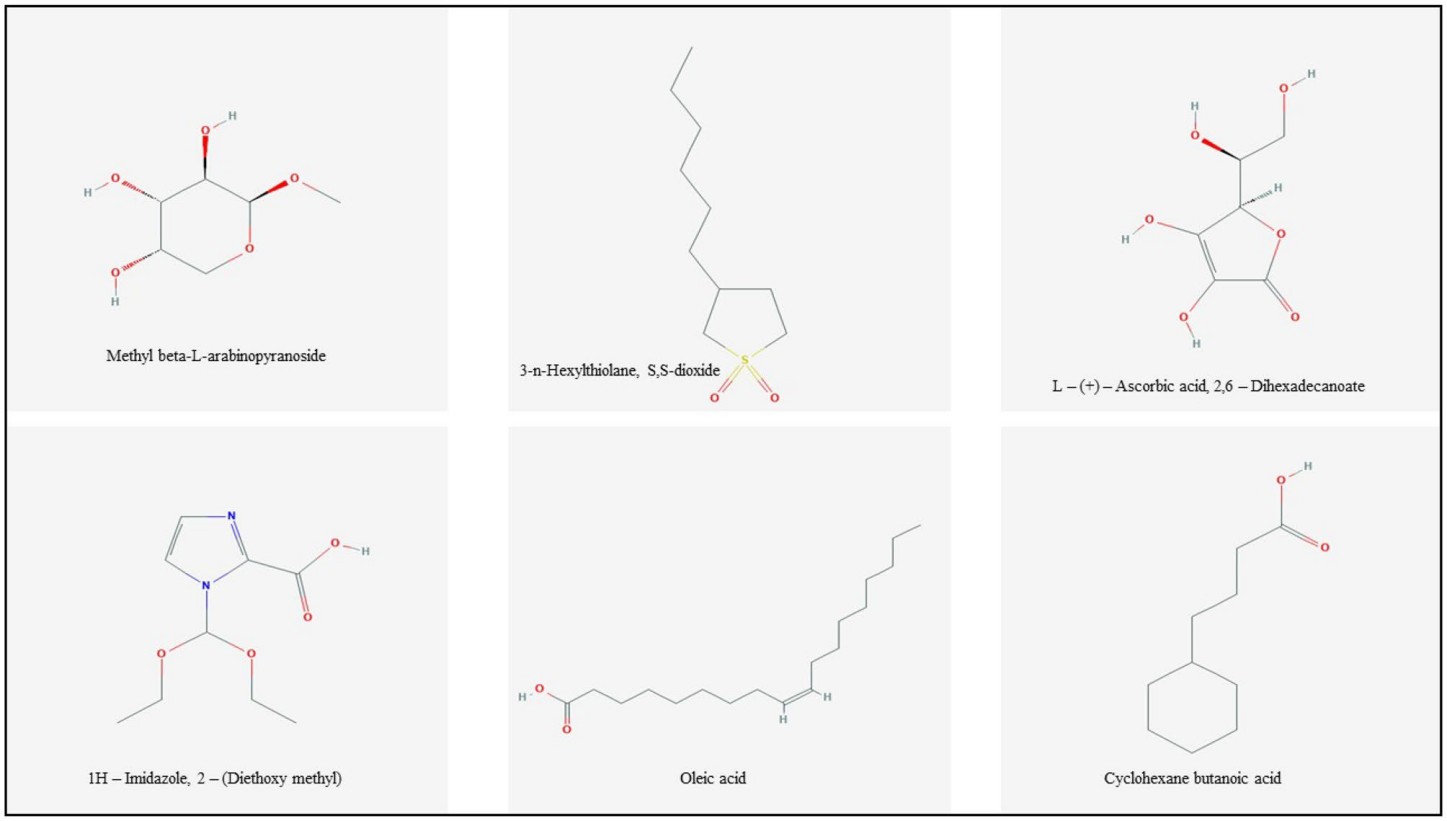
Structure of bioactive compounds identified from *P. timoriana* and used for the analysis of molecular docking. (Photo courtesy: www.pubchem.ncbi.nlm.nih.gov).

**Figure 9:**
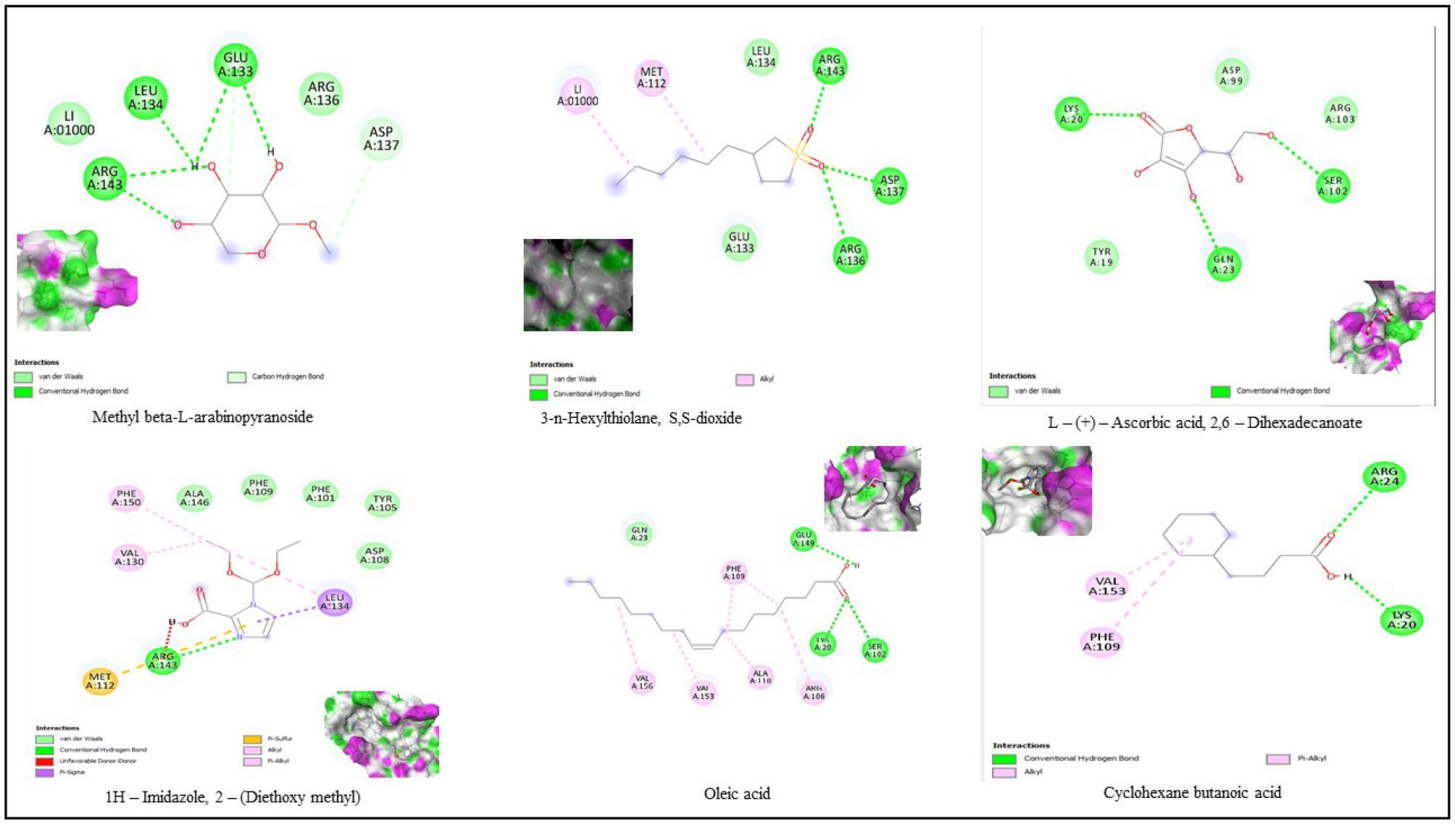
The bioactive compounds from *P. timoriana* extracts docked with BCL-2 protein.

**Figure 10:**
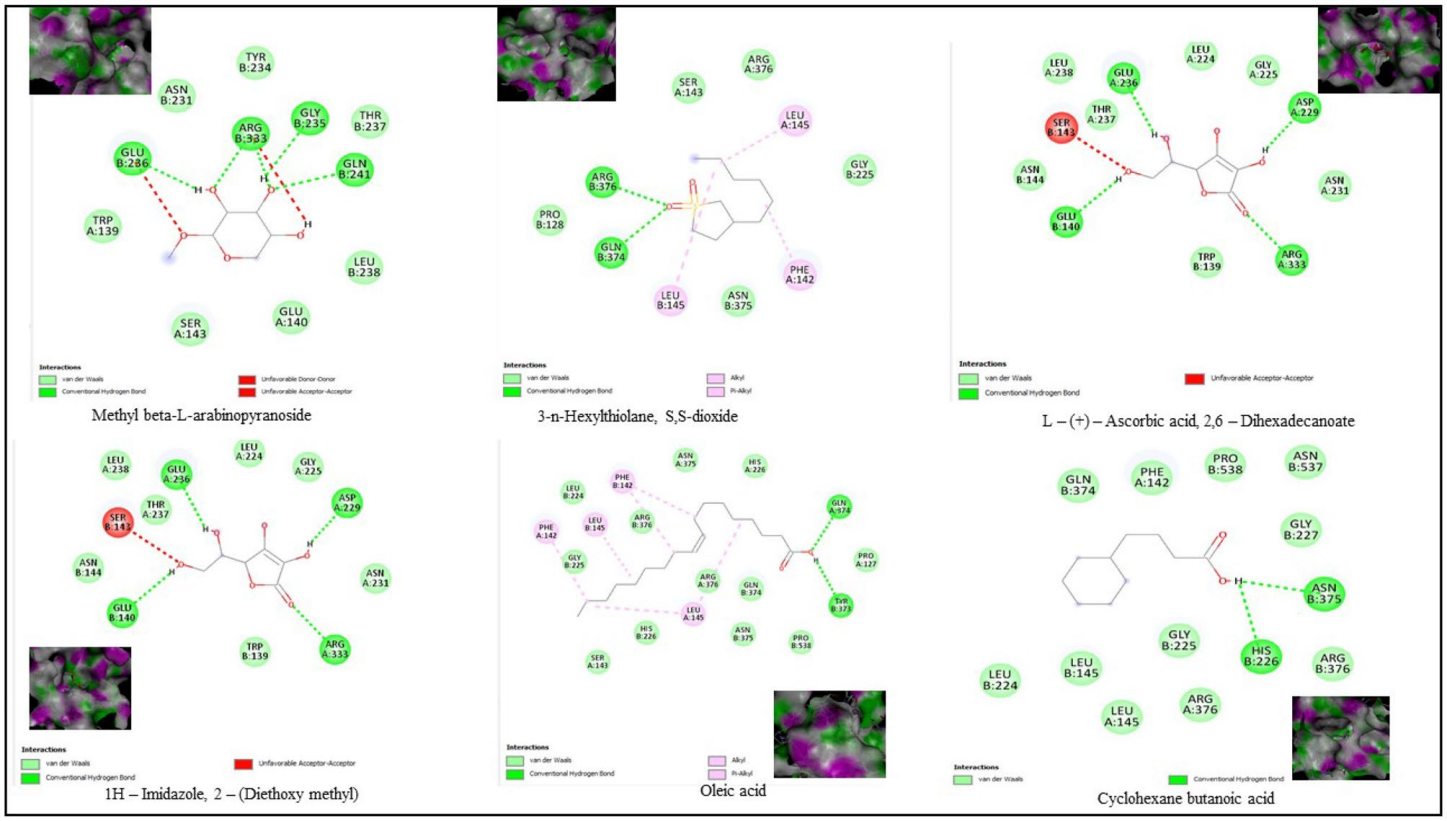
The bioactive compounds from *P. timoriana* extracts docked with COX-2 protein.

Structures of the bioactive compounds are shown in Fig. 8. The binding analysis between COX-2 protein and ligands, BCL-2 and ligands revealed that the binding pattern varied with the nature of the ligands. The docking results of bioactive compounds are shown in Figs. 9 and 10. And the docking results are represented in the form of minimum binding energy values (Tables 9 and 10).

**Table 9:** Molecular docking score for bioactive compounds of *P. timoriana* with BCL-2.

Sl. No	Ligand	Docking score	No of hydrogen bonds formed
1	Methyl beta-L-arabinopyranoside	− 4.3	4
2	3-n-Hexylthiolane, S,S-dioxide	− 6.3	3
3	L-(+)-Ascorbic acid, 2,6-Dihexadecanoate	− 4.4	3
4	1H-Imidazole, 2-(Diethoxy methyl)	− 4.8	1
5	Oleic acid	− 5.5	2
6	Cyclohexane butanoic acid	− 4.5	0
7	Paclitaxol	− 10.8	3

**Table 10:** Molecular docking score for bioactive compounds of *P. timoriana* with COX-2.

Sl. No	Ligand	Docking score	No of hydrogen bonds formed
1	Methyl beta-L-arabinopyranoside	− 5.6	4
2	3-n-Hexylthiolane, S,S-dioxide	− 5.7	2
3	L-(+)-Ascorbic acid, 2,6-Dihexadecanoate	− 6.0	4
4	1H-Imidazole, 2-(Diethoxy methyl)	− 5.8	4
5	Oleic acid	− 5.3	2
6	Cyclohexane butanoic acid	− 5.7	2
7	Isoxicam	− 9.3	3

## Discussion

The indigenous people of NE India commonly consume this plant because of its nutritive and medicinal benefits. The plant parts especially the bark, fruit skin, leaf, seeds are used for the treatment of various diseases^17^. The tender pods (young and mature), flowers, capitulum and mature seeds have also been traditionally used as supplementary food sources in this region. In *P. timoriana*, flowers, capitulum, fruits and seeds are consumed as either raw as salads or boiled with water as vegetables that contributes to the health benefits. Tree bean is also used as a supplementary food source and provides quality food for human as well as livestock, and is touted as a multifunctional vegetable that serves as valuable and dependable income-generating products for farmers of NE India. Ethnobotanical data hinted *P. timoriana* as a multi-functional plant that had nutritional as well as medicinal values and also provided that *P. timoriana* likely contained bioactive compounds which could be used in antidiabetic, anti-hypersensitive, anti-inflammatory, anti-stomachache and antimicrobial medicines.

Flavonoids are shown to be effective against cancer cells by inactivating or inhibiting carcinogens, anti-proliferative, induced apoptosis and cell cycle arrest^43^. Terpenoids also have several curative properties like anti-cancerous, anti-parasitic, anti-allergic, and anti-inflammatory^44^. Likewise, saponins and alkaloids have been reported to have anticancer properties especially against colon cancer, and saponins can also induce growth inhibition and apoptosis^45^. Traditionally, tannins are also used for the treatment of diarrhoea, haemorrhage, detoxification etc.^46,47^. Studies have shown that flavonoids and phenolic compounds present in plants have a wide variety of biological effects including antioxidant, anti-inflammatory, antimicrobial, anti-angiogenic, anticancer, anti-allergic properties etc.^48^.

In our study, a significantly higher flavonoid content was observed in the methanolic extract. Flavonoids are naturally occurring compounds and water-soluble polyphenolic compounds responsible for coloration in vegetables, fruits and herbs. The biological function of flavonoid is extensive and are used as anti-cancer, anti-inflammatory, anti-allergic, anti-thrombotic, tumour inhibitor, anti-viral agents, in heart diseases and also has a large effect in the lower intestinal tract^49–51^. They also possess antioxidant activities that can prevent oxidative cell damage.

Plants produce phenolic compounds as secondary metabolites during normal development^52^. Phenols are bioactive compounds that acquire antioxidant potential responsible for health benefits. Phenolic compounds present in the plant can play various roles and are often used in skin infection, wounds treatment etc.^53^. Plants with high phenol contents are usually targeted by pharmacists the world over for the treatment of various diseases^54^. The total phenol content was considerably high and hence the extract could have the ability to treat many diseases. Hence, this plant is of special interest for further investigations.

Free radicals and other reactive oxygen species (ROS) have been implicated in various diseases like neurodegenerative diseases, cancer and cardiovascular diseases. Bioactive compounds present in plants have long been known to possess antioxidant, anti-allergic, anti-inflammatory, anti-viral, anti-proliferative and anti-carcinogenic properties because of which a lot of attention is given to the evaluation of the antioxidant capacity of foods, medicinal plants and other nutritional antioxidant supplements^55^. The DPPH is a stable free radical that is commonly used to analyze free radical scavenging activities of antioxidant^56^. Antioxidant molecules can quench DPPH and transform them into colorless products^57^. A lower value of IC_50_ (half maximal inhibitory concentration) designates the stronger ability to scavenge free radicals whereas the higher value of IC_50_ designates weaker scavenging activity, as more scavengers are required to achieve 50% scavenging reactions. The phenolic compounds act as primary antioxidants or free radical scavengers^58^. They contain hydroxyls that are responsible for the radical scavenging effect due to redox properties^59^. Hence, high free radical scavenging activity in the current investigation could be attributed to high phenolic and flavonoid contents in the plant extracts. Suggestively, *P. timoriana* could be used as a source of antioxidants for pharmacological preparations.

The antioxidant capacity of the extracts was also determined using the phosphomolybdate assay, which was based on reducing Mo (VI) to Mo (V) by the test sample and the subsequent production of green phosphate compounds with absorbance at 765 nm^60^. Various flavonoids and related polyphenols have been found to contribute considerably to the phosphomolybdate scavenging potential of herbal plants in recent research^61^.

The ABTS radical scavenging assay uses a reaction of ABTS and potassium persulfate to produce a blue ABTS^+^ chromophore^60^. The scavenging activity was found significantly high in the pod part which was comparable to the standard, Ascorbic acid. Interestingly in all the antioxidant assays, the scavenging activity was found the highest in the pod part that also had significantly high flavonoid and phenol contents compared to other parts. Furthermore, from the GC–MS analysis, the bioactive compound ascorbic acid was also detected from the pod and that could be the reason for high antioxidant potential in the pod.

**Figure 11:**
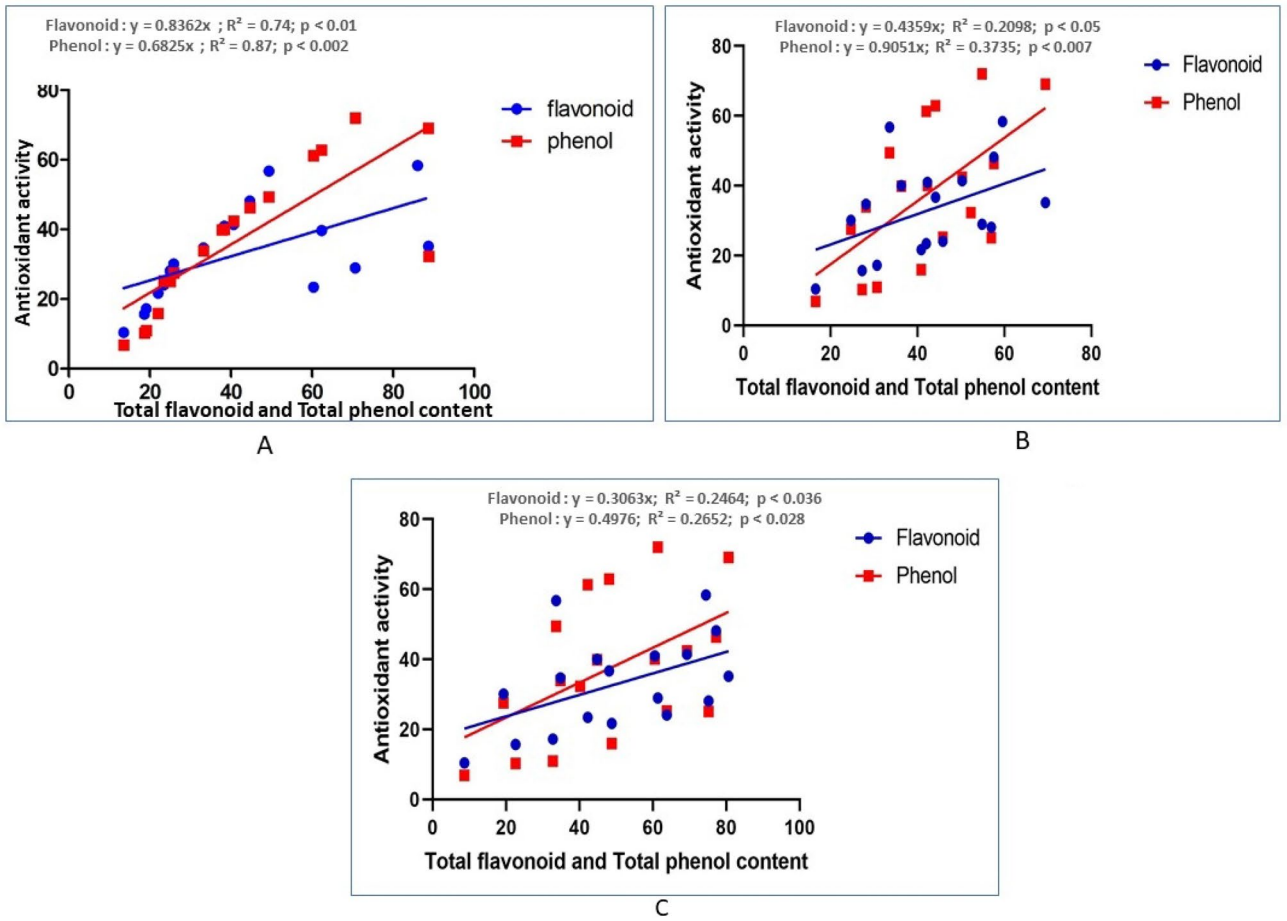
Correlation values of antioxidant versus total phenol and flavonoid content. (**A**) DDPH antioxidant activity; (**B**) ABTS antioxidant activity; (**C**) Phosphomolybdate antioxidant activity.

Phenol and flavonoid molecules are essential antioxidant compounds that are responsible for oxidizing free radicals due to their capability to hand out hydrogen atoms to free radicals. A linear correlation of total phenolic and flavonoid contents with an antioxidant potential of plant extracts is already established^62^. The correlation of total phenolic and flavonoid content with the antioxidant potential of *P. timoriana* is shown in Fig. 11. High correlations between DPPH antioxidant activity and total phenol (y = 0.682x; R^2^ = 0.87, *p* < 0.002) and total flavonoid (y = 0.839x; R^2^ = 0.74, *p* < 0.01), ABTS antioxidant activity and total phenol (y = 0.9051x; R^2^ = 0.3735, *p* < 0.007) and total flavonoid (y = 0.4359x; R^2^ = 0.2098, *p* < 0.05) and phosphomolybdate antioxidant activity total phenol (y = 0.4976x; R^2^ = 0.265, *p* < 0.028) and total flavonoid (y = 0.3063x; R^2^ = 0.246, *p* < 0.036) and were observed at a 95% confidence level. Accordingly, at correlation coefficients, it is attainable to speculate that phenol and flavonoid compounds have played significant roles in the antioxidant activities of the plant extracts.

Infectious diseases caused by bacterial agents pose a major threat to public health worldwide^63^. And the rise of antimicrobial resistance and toxicity problems, on the other hand, has limited the use of antimicrobial agents^64^. Antibiotics have safety and efficacy limits and due to similar toxicity and efficacy, biological research on antimicrobial functions of plants should be expanded^65^. The bioactive compounds present in plants such as alkaloids, flavonoids, tannins, phenol, saponins etc. are their secondary metabolites that act as defense mechanism against microorganisms, insects and other herbivores^66^ and the presence of these compounds in tree bean could be responsible for the observed antimicrobial property. Among them, plants in response to microbial infections synthesize flavonoids and it is a successful antimicrobial substance against a broad range of microbes^67^. Phenols are widely present in secondary products of medicinal plants as well as in many edible plants and have the potential of antioxidants and free radical scavengers^68,69^. Saponins also have antimicrobial properties as they can induce protein and enzyme leakage from cells^70^. Tannins bind to proline-rich proteins, thus, stopping them from being synthesized^71^. It was also reported that tree bean could inhibit pathogenic bacterial growth such as *E. coli, V. cholera, S. aureus*, *B. cereus Streptococcus fecalis*^21,72,73^. Similarly, in the present study, different *P. timoriana* extracts were seen to be effective against many cosmopolitan as well as pathogenic microorganisms including *E. coli* (urinary tract infections and diarrhoea), *B. subtilis* (food poisoning), *P. aeruginosa* (urinary tract, respiratory system infection) and *B. pumilus* (foodborne disease, severe sepsis of neonatal infants etc.). It is interesting to note that the multi-drug resistant *P. aeruginosa* and *B. subtilis* strains showed more sensitivity to the tested extracts.

Preliminary antibacterial investigations using the well diffusion assay revealed an intriguing antimicrobial activity profile against both gram-positive and gram-negative bacteria. Then, a quantitative investigation was done to determine the total bactericidal activity of extracts using MIC. The plant part extracts showed moderate to excellent efficacy against bacterial strains with MIC values ranging from 2.19 mg/ml to 7.3 mg/ml. It has been proposed that all plant species with MIC values of up to 8 mg/ml have at least some inhibitory action, and any dose above this should not be regarded as effective^74^. Among the studied extracts, the pod appeared to have the majority of the moderate inhibitors with an intriguing bactericidal effect against *E. coli* (2.19 mg/ml).

Our findings support the use of tree bean in folk medicine for the treatment of several infectious diseases. It is opined that *P. timoriana* species can be a good source for antibacterial drugs against gram-positive and gram-negative bacteria, especially against multi-resistant microorganisms. However, isolation of these bioactive antibacterial compounds and detailed characterization of them should provide a deeper understanding of drug discovery and development.

The functional groups can be used in different pharmaceutical products such as anti-cancers, anti-ulcers, anti-inflammatory, anti-oxidants, antimicrobial etc.^75,76^. Hence, the functional groups present in the bioactive compounds can have diverged applications including antimicrobial, anti-cancer and anti-inflammatory properties etc. FTIR spectroscopy is a reliable and sensitive method for the detection of bioactive compositions. Our analysis showed clear discrimination between the plant parts examined, depicting great variations. It was also able to determine the bioactive compounds that could be used as herbal medicines for various diseases.

Most of the compounds identified are known to possess a variety of pharmacological activities. The higher total phenol and flavonoid contents, the significant potential of antioxidant and antimicrobial activities are attributed to the presence of these bioactive compounds (Table 9). From the chromatogram, 8 compounds (Beta-L-Arabinopyranoside Methyl, Heptanoic acid, Octanoic acid, Z, Z-6-28—Heptatriactontadien-2-one, 2- Pentacosanone, Methyl Stearate, Palmitoleic acid, Dihexadenyl phosphate) were found in all the plant parts and the biological significance of these compounds are shown in Table 6. The compounds -Phthalic Acid, 3-Methyl-2-(2-Oxopropyl) Furan, Choloroacetic acid, tetradecyl ester, 1-Cyclohexane-1-Methanol, 4-(1-Methyl ethenyl), Formate etc. possessed antioxidant potentials. Interestingly, among all extracts, L-(+)-Ascorbic acid, 2,6-Dihexadecanoate was found only in the pod. This could be the reason for the highest antioxidant IC_50_ and free radical scavenging activity by the pod. The compounds such as Oleic acid, 3-Methyl-2-(2-Oxopropyl) Furan, 3-n-Hexylthiolane, S, S-dioxide, 3-n-Hexylthiolane, S, S-dioxide, Nonanoic acid, Oxirane, hexadecyl etc. were shown to possess significant antimicrobial potential hence higher antimicrobial activity was shown by the extracts. Compounds identified previously from *P. biblosa* such as 1,2,4-Benzenetriol, linoleic acid, 6-Octadecanoic acid, oleic acid, palmitoleic acid, 9-Decanoic acid, cyclohexane butanoic acid, N-Decanoic acid were also detected in our study^19^. All the identified compounds belonged to various chemical groups and a majority of them have been confirmed to have significant biological activities^19^. And some compounds are yet to be defined in detail. However, further investigation on isolation, characterization of the compounds from plant extracts is sought to verify their various pharmacological significance.

Computational advances played a significant influence in the drug development process. Virtual screening approaches are frequently and widely utilized to minimize the cost and time of drug development. Molecular docking is a technique that is used to discover novel ligands for proteins structure and plays a significant role in structure-based drug design^77^. The relationship between the compounds and the receptor plays a vital role in the formulations of drugs. Natural products can help cure a wide variety of human ailments, including cancer, inflammatory, etc. and various medicines are derived from natural products, e.g., an anticancer drug, paclitaxel (taxol) is derived from extracts of *Taxus brevifolia.* Plant-based medicines are the best potential options, given the rise of drug resistance in various diseases, and the negligible side effects of traditional treatments^78^. We investigated the binding capability of bioactive compounds from *P. timoriana* with key anticancer and anti-inflammatory targets in this study since various ailments had been traditionally treated with *P. timoriana*. The higher negative docking score represented a high binding affinity between the receptor and ligand molecules, showing the higher efficiency of bioactive compounds. The docked ligand scores for anticancer ranged from -4.3 to -6.3 and from -5.3 to -6.0 for anti-inflammatory. In the present study, 3-n-Hexylthiolane, S, S-dioxide, and L-(+)-Ascorbic acid are the lead compounds showing the highest docking score among the subjected bioactive compounds, which also exhibited antibacterial, antioxidant potentials. The docking analysis also revealed that diverse energy sources were consistent and contributed to the overall strength of 3-n-Hexylthiolane, S, S-dioxide, and L-(+)-Ascorbic acid binding with each target protein. Methyl beta-L-arabinopyranoside had the lowest score while 3-n-Hexylthiolane, S, S-dioxide had the highest score as anticancer agents. The anti-inflammatory potential was found the highest in L-(+)-Ascorbic acid, 2,6-Dihexadecanoate and lowest in Oleic acid. Nonetheless, all the compounds were in considerable range as anti-inflammatory and anti-cancer therapeutic agents. Our study shows that the selected bioactive compounds can efficiently bind to the receptors and molecular docking can be successfully used in finding BCL-2 and COX-2 inhibitors from *P. timoriana* extracts. Interestingly, the presence of diverse bioactive compounds supports the traditional practitioners’ use of these plant parts for the treatment of various diseases. Hence, the studied bioactive compounds have the potential to be used as anticancer and anti-inflammatory agents against these diseases. To the best of our knowledge, this is the first report that bioactive compounds from underutilized, ethnobotanically important *P. timoriana* are subjected to molecular docking for screening their pharmacological potentials.

## Conclusions

The present study was focused on identification of various bioactive compounds from the edible parts of *P. timoriana* for the first time by FTIR and GC–MS analysis. The bioactive compounds are responsible for various therapeutic and pharmacological properties. The study also provided the evidence of *P. timoriana* extracts for its antioxidant and antimicrobial potentials. The bioactive compounds Methyl beta-L-arabinopyranoside 3-n-Hexylthiolane, S,S-dioxide, L-(+)-Ascorbic acid, 2,6-Dihexadecanoate, 1H-Imidazole, 2-(Diethoxy methyl), Oleic acid, Cyclohexane butanoic acid showed promising binding affinity towards the two target proteins in the molecular docking studies. From our findings, the plant may enable us to develop reliable and effective drugs against various diseases. Further investigations to analyze its bioactivity and clinical trials are necessary for discovery of new drugs formulations.

## Methods

### Collection of plant material

**Figure 12:**
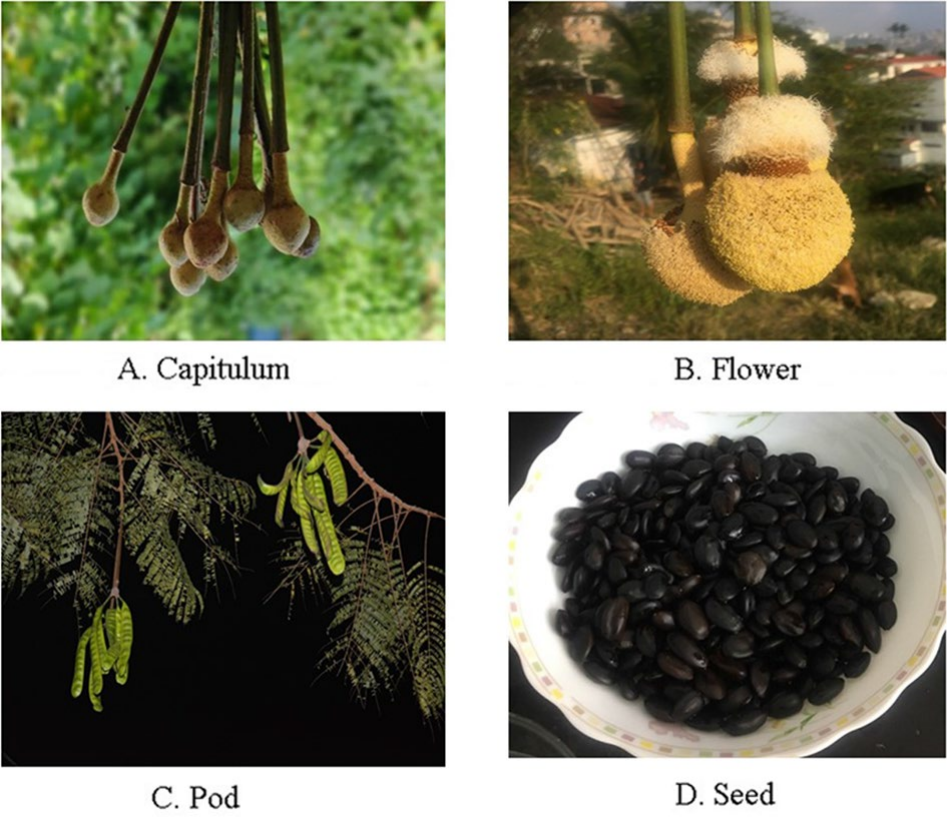
Edible parts of *P. timoriana* (**A**. Capitulum, **B**. Flower, **C**. Pod, **D**. Seed).

Edible parts of *Parkia timoriana* viz. flower, capitulum, pod and seed (Fig. 12) were collected from Aizawl, Mizoram, India. The collected samples were brought to the Department of Botany, Mizoram University for further analysis. Identification of the collected specimen were done following published literature^79,80^. The specimens were also deposited in the Herbarium of the Department of Botany, Mizoram University (Voucher no. MZUBOT0100) and identified and confirmed by Dr. Khomdram Sandhyarani Devi, Plant Taxonomist, Department of Botany, Mizoram University. The ethnobotanical survey was conducted in the Aizawl district of Mizoram. The study was based on personal interviews with local herbal medicine practitioners and other knowledgeable local people and published literature.

### Bioactive compounds analysis

A 50 g of air dried powdered edible part was extracted with 500 mL of methanol using a Soxhlet apparatus at moderate temperature of 30–45 °C to avoid disruption of thermolabile compounds for 25 cycles. The extract was then concentrated at 50 °C until it formed a paste. The concentration of each sample was adjusted to 100 µg/mL using methanol. Presence of various phytochemicals: alkaloids, saponins, flavonoids, tannins and terpenoids from each extract were estimated after Nwankwo and Ukaegbu-Obi^81^.

### Determination of Total Phenolic Content (TPC)

Total phenol was determined using the Folin-Ciocalteu reagent method^82^ with slight modifications. A 100 μl plant extract sample was mixed with 0.1 ml Folin-Ciocalteu reagent (1 N) and incubated at room temperature. Then, 5 ml of Na_2_CO_3_ was added and incubated at room temperature for 30 min. Total phenolic content was determined using a UV–VIS spectrophotometer (Biospectrometer, Eppendorf, Germany) at 760 nm. Gallic acid was used as standard and total phenol was expressed as gallic acid equivalent (mg/g of the extracted compound).

### Determination of Total Flavonoid Content (TFC)

The total flavonoid content was determined using the aluminium chloride calorimetric method^83^. Briefly, 1 ml extract was mixed with 1 ml methanol, 0.5 ml aluminium chloride (1.2%) and 0.5 ml potassium acetate (100 mM) and incubated at room temperature for 30 min. The absorbance was measured at 415 nm, and quercetin was used as standard. The total flavonoid content was expressed as quercetin equivalent (mg/g of the extracted compound).

### DPPH radical scavenging activity

The antioxidant activity of the extract was determined using the 2,2-diphenyl-1-picrylhydrazyl (DPPH) radical scavenging method^84^. To 50 µl of 10–100 µg/mL plant extract, 2 ml DPPH was added and kept in dark at room temperature for 30 min. Then, 1 ml methanol and 2 ml DPPH was used as negative control while methanol solution was used as a positive control. Then the absorbance was measured at 517 nm. The percentage DPPH radical scavenging activity (%RSA) was calculated as:

$$\%{\text{RSA}} = 100 \times ({\text{absorbance of control}} - {\text{Absorbance of sample)}})/{\text{Absorbance of control}}$$

where control is 2 ml of DPPH + 1 ml of methanol.

### ABTS radical scavenging assay

The 2,2’–azinobis (3-ethylbenzthiazoline-6-sulphonic acid) ABTS radical cation decolorization test was used to assess the free radical scavenging activity of plant extracts^85^. The ABTS^+^ cation radical was created by reacting 7 mM ABTS in water with 2.45 mM potassium persulfate (1:1) and kept in the dark at room temperature for 14–16 h before use. After diluting the ABTS^+^ solution with methanol, the absorbance of 0.700 at 734 nm was obtained. The absorbance was measured 30 min after the addition of 10 µl of plant extract to 3.990 ml of ABTS solution. In each experiment, a suitable solvent blank was run. All the tests were repeated at least three times and ascorbic acid was used as standard. The ABTS scavenging effect (%) was calculated as:

$${\text{\% inhibition}} = {\text{Absorbance of Control}} - {\text{Absorbance of sample)}} \times 100/{\text{Absorbance of control}}$$

where control is ABTS solution with methanol.

### Phosphomolybdate assay

Using ascorbic acid as a reference, the total antioxidant capacity of the plant extract was measured using phosphomolybdate method^86^. A total of 1 ml of reagent solution (0.6 M sulphuric acid, 28 mM sodium phosphate, and 4 mM ammonium molybdate) was combined with 0.1 ml of plant extract. The tubes were incubated for 90 min at 95 °C water bath. The absorbance was measured at 765 nm against a blank after the samples had been cooled to room temperature. The antioxidant capacity was calculated as:

Antioxidant effect (%) = (Control absorbance – sample absorbance)/ (control absorbance) × 100

where control is reagent + methanol.

### Antimicrobial activity

The antimicrobial activity of *P. timoriana* extract was measured using the agar well diffusion method against four bacterial strains- *Bacillus pumilus* (ATCC14884), *Bacillus subtillis* (ATCC11774), *Escherichia coli* (ATCC11229) and *Pseudomonas aeruginosa* (ATCC9027), Rifampicin was used as a positive control. The zone of inhibition of each bacterium was measured using Antibiotic Inhibition Scale (HiMedia, India). The antimicrobial activity (x) was calculated and characterized after Obdak et al.^87^ Then, the antimicrobial activity (x) was designated as slight (x < 4 mm diameter), medium (x = 4–8 mm), high (x = 8–12 mm) and very high (x > 12 mm).

### Preparation of McFarland standard

To determine bacterial density, a McFarland number 0.5 standard was prepared by combining 9.95 ml of 1% H_2_SO_4_ in distilled water and 0.05 ml of 1% BaCl_2_ in distilled water^90^. The mixture was kept in an airtight bottle and used to compare bacterial suspensions as needed.

### Minimum inhibitory concentration (MIC)

For determination of mimimum inhibitory activity of the plant extracts, the broth micro dilution technique was used using 96- well microtiter plate^88^. Different concentrations of the extracts (0.5 mg/ ml to 20 mg/ml) were prepared with methanol. Overnight grown culture of the bacteria was used for inoculation. Plates were incubated at 37 °C at 120 rpm shaking for 24 h. Growth inhibition was checked by taking the absorbance at 600 nm in a UV–VIS spectrophotometer (Multiscan™ GO, Thermo Scientific,USA) along with the controls. Then, IC_50_ values were expressed as the concentration of plant extract necessary to produce a 50% reduction of the bacterial growth.

### FT-IR analysis

Functional groups present in the *P. timoriana* extract were identified using Fourier transformed infrared spectroscopy (Shimadzu IRAffinity-1S, Japan) for frequency ranging from 400–4000 cm^-1^ following the manufacturer's instruction.

### GC/MS analysis

#### Sample preparation

A 100 µl of methanolic plant extract was dissolved in 1 ml of methanol solvent. The solution was stirred vigorously using vortex stirrer for 10 secs and filtered using 0.2 micron membrane filter. Then, the clear extract was used for GC–MS analysis.

The identification of compounds was carried out by using GC–MS (Clarus 690, Perkin-Elmer, USA) (Auto system XL) fitted with a capillary column (123.5 m x 678 μm) coupled to a mass detector Turbo mass gold -Perkin Elmer Turbo mass 5.1 spectrometer with an Elite—1 (100% Dimethyl poly siloxane), 123.5 m x 678 μm of the capillary column. The instrument was fixed at an initial temperature of 40 °C and ramp 5 °C/min to 115 °C, hold 5 min, ramp 5 °C/min to 140 °C, hold 5 min, ramp 2 °C/min to 210 °C, hold 8 min, and maintained at this temperature for 3 min. At the end of this period, the oven temperature was increased up to 250 °C, at the rate of an increase of 5 °C/min, and maintained for 9 min. Injection port temperature was ensured at 250 °C and Helium flow rate at 1.5 ml/min. The ionization voltage was set at 70 eV. The samples were injected in split mode as 10:1. Mass spectral scan range was set at 50–800 (m/z). The ion source temperature was maintained at 230 °C and the Interface temperature at 240 °C. The MS start time was set at 3 min, and the end time was 75 min with a solvent cut time of 3 min. The spectrum obtained of volatile compounds detected through GC–MS were compared and matched with NIST 17 (National Institute of Standard and Technology) online library Ver. 2.3.

### Molecular docking analysis

Molecular docking is used to find the best-fit orientation of ligand and protein. The bioactive compounds from *P. timoriana* were selected for molecular docking analysis. The target proteins BCL-2 and cyclooxygenase (COX-2) were docked using selected bioactive compounds using BIOVIA, Discovery Studio (version 2021) and AutoDock Vina software and binding energies were calculated. The ligands and the target proteins were prepared following the standard procedure of protein and ligand preparation and the files were submitted to AutoDock vina. The obtained binding energy and binding contacts of each ligand, and the docked data were analyzed using Discovery Studio Visualizer.

#### Preparation of ligand

Six compounds were predicted based on Lipinski’s rule of five^89^ for the docking analysis. The chemical structures of the selected bioactive compounds were retrieved through the PubChem compound database at NCBI (http://pubchem.ncbi.nlm.nih.gov/).

#### Target protein retrieval

Two proteins, BCL-2 (PDB ID: 1PXX) for anticancer and cyclooxygenase-2 (COX-2) (PDB ID: 1K3K) for anti-inflammatory were selected for their interactions with the bioactive compounds from *P. timoriana*. Expression of BCL2 is thought to be the cause of follicular lymphoma. And the COX-2 plays a significant role in the modulation of inflammation. The 3D X-ray crystal structure of the proteins was retrieved from Protein Data Bank (PDB).

#### Target protein preparation

The Discovery Studio software was used to process and prepare the proteins and convert raw PDB structure into prepared protein models. And X-ray crystal structures of the proteins were prepared by removing the water molecules present in the structures. Then, Discovery Studio software was used to analyze protein structure, hydrogen bond interactions and non-bond interactions of ligands with the active site residues and generations of high-quality images.

#### Docking

The prepared ligands and target proteins were analyzed using AutoDock Vina to perform the docking. The various conformations for ligand in the docking procedure were generated and the final energy refinement of the ligand pose was performed. The docking score of the best pose into the target proteins for all the tested bioactive compounds was calculated.

### Statistical analysis

All the experiments were carried out in triplicate, and the findings were reported as mean ± SD. All the data and the linear regression coefficient (R^2^) for total flavonoid and phenolic content with antioxidant activity was analyzed using Graph Pad Prism Version 5. P-value < 0.05 was considered significant.

### Ethical approval and informed consent

 Authors declare that all methods were carried out in accordance with relevant guidelines and regulations. Authors confirm that all experimental protocols were approved by the Institutional Human Ethics Committee, Mizoram University.Authors also confirm that informed consent was obtained from all subjects.

### Material collection

 For collection of plants, all relevant permits or permissions have been obtained. The study also complies with local and national regulations.
